# Sea-level proxies in Holocene raised beach ridge deposits (Greenland) revealed by ground-penetrating radar

**DOI:** 10.1038/srep46460

**Published:** 2017-04-19

**Authors:** Lars Nielsen, Mette Bendixen, Aart Kroon, Mikkel Ulfeldt Hede, Lars B. Clemmensen, Ronny Weβling, Bo Elberling

**Affiliations:** 1 Department of Geosciences and Natural Resource Management, University of Copenhagen, Øster Voldgade 10, 1350 Copenhagen K, Denmark; 2 Center for Permafrost (CENPERM), Department of Geosciences and Natural Resource Management, University of Copenhagen, Øster Voldgade 10, 1350 Copenhagen K, Denmark; 3 Department of Prehistoric and Historical Archeology, University of Vienna & Crazy Eye, Geoinformatics and Digital Archaeology, Franz-Klein-Gasse 1, 1190 Vienna, Austria

## Abstract

Identification of sea-level proxies is important for reconstruction of past sea-level variation. Methods for reconstructing Holocene relative sea-level curves are crucial for quantification of the impact of Greenland ice thickness variation on global sea level and vertical land movement. Arctic beach ridges constitute important potential archives of sea-level variation. However, their surface morphology may have undergone modification since deposition due to freezing/thawing processes and erosion, and their morphology may therefore not be trustworthy for sea-level reconstruction. Therefore, geophysical imaging is used to examine the internal structures of the beach ridges and to define a sea-level proxy unaffected by surface processes. The GPR reflections from study sites in West and South Greenland show deposition of beachface deposits and upper shoreface deposits; the contact between steeply dipping beachface reflections and less-dipping shoreface reflections is used as sea-level proxy. Numerous points are identified along GPR transects facilitating reconstruction of relative sea-level variation of hitherto unprecedented resolution. Erosional events and deformation caused by freezing/thawing processes are clearly delineated. The approach constitutes a solid base for reconstruction of relative sea-level curves affected by a well-defined vertical land movement history since the studied beach ridge systems represent long time intervals and only relatively small spatial extents.

Curves of relative sea-level variation during the Holocene in Greenland and the surrounding Arctic are critical for our understanding of past and present absolute sea-level change as well as to reveal the impact of vertical land movement due to ice sheet thickness variations or other (e.g. tectonic) effects^1^,^2^,^3^. Several studies of Holocene sea-level changes in Greenland and the surrounding Arctic have been made based on geological and geographical investigations of e.g. isolation basins, beach ridge morphology, salt marsh deposits, and mapping of paleo-shorelines^2^,^4^,^5^,^6^,^7^,^8^,^9^,^10^,^11^,^12^,^13^. Radar methods have been extensively employed in studies of ice sheet thickness variation in Greenland^14^, but no extensive studies of beach deposits and relative sea-level based on GPR have been reported from this area.

The formation of gravelly berm ridge deposits is mainly determined by high-energy wave processes during onshore storms with associated elevated water levels, and they typically form on the backshore at the upper limit of the swash excursion during the storm event^15^,^16^,^17^,^18^. Newly formed berm ridges can be transformed into beach ridges by coastal progradation, and they can ultimately form raised beach ridge systems in areas with land uplift and sufficient sediment supply^19^,^20^. The morphology and internal structure of beach ridges thus constitute important archives for understanding not only coastal evolution, but also storminess and relative sea-level variation in the past^18^,^19^,^20^,^21^,^22^,^23^,^24^.

GPR profiling of raised, fossil beach ridges forms an optimal basis for reconstruction of relative sea-level variation of the past^19^,^20^,^25^,^26^ and for imaging sedimentary structures indicative of climatic changes^24^. However, GPR imaging and mapping of modern beach deposits near present coast-lines may be challenged by salt-water intrusion causing dampening of the electromagnetic signals^23^.

The accuracy of relative sea-level curves is mainly determined by the type and abundance of sea-level markers found in the geological record and in surface morphology, and by the accuracy of dating. The first factor is addressed in this study. Besides, the use of a GPR-based method for mapping high-resolution sea-level markers in the Greenlandic environment is demonstrated and discussed. Markers of-sea-level have been identified in GPR data collected in fossil beach deposits in Japan^16^ and in the micro tidal environment of southwest Scandinavia^23^. Such markers allow for detailed reconstruction of Holocene sea-level variation^19^,^20^,^26^.

High-resolution ground-penetrating radar (GPR) reflection images are collected across raised beach ridges at Tuapaat (Disko Island, West Greenland), and at Igaliku and Qassiarsuk in South Greenland (Fig. 1). These beach ridge systems have previously been identified and described from geomorphological and sedimentological observations^4^,^6^,^27^. Tuapaat is located in the vicinity of the Jacobshavn Isbræ glacier (opposite side of Disko Bay); an area which is currently undergoing significant vertical land movement as measured by GPS^28^. The localities at Igaliku and Qassiarsuk are thought to have been affected by complex glaciation histories during the Holocene^4^. The GPR reflection images are interpreted with a focus on understanding the internal beach ridge layering and structure and identify possible markers of past sea-level variation. The robustness of the methodology with respect to sea-level marker identification in areas affected by erosion and freeze/thaw processes is described and discussed. Moreover, the potential advantage of using the presented approach under Greenlandic conditions as compared to traditional, extensively used methods such as, for example, isolation basin studies, geomorphological mapping of raised beach deposits, and identification of marine limits is also discussed. Thus, the focus of this report is on the application of the GPR method for imaging internal structures of Greenlandic beach ridge plains that may be used as relative sea-level indicators, but not on the establishment of relative sea-level curves.

## Study sites

**Figure 2:**
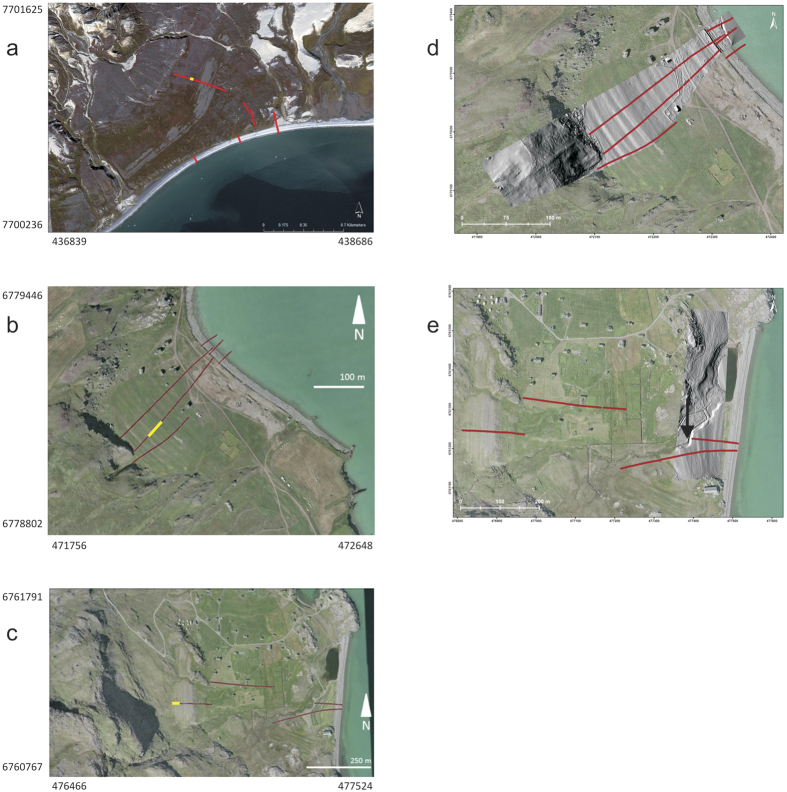
Satellite images showing field sites at Tuapaat (**a**), Qassiarsuk (**b**), and Igaliku (**c**). Differential GPS recording along GPR lines are shown. Subsection of the GPR sections shown in Fig. 3 and the Supplementary Material are indicated by yellow lines. Note that in some cases the differential GPS surveys were continued into the water beyond the coast line in order to capture sub-sea-level topography. The GPR lines were terminated just before the position of the coast line at the time of recording. Digital elevation models derived from kite aerial photography showing topography of parts of the elevated beach ridge plains at Qassiarsuk (**d**) and Igaliku (**e**). The kite aerial elevation data cover the interval from the present coast line to ~34 m elevation at Qassiarsuk and to an elevation of ~8 m at Igaliku. Black arrow points towards outcrop where the photograph of Fig. 4 was taken. Map corner coordinates are in UTM. The high-resolution satellite images are generated using Google Earth. The satellite images are provided on August 30, 2012 (Tuapaat; data provider: Asiaq, image by NASA), May 5, 2013 (Qassiarsuk; data provider: CNES/Astrium), and August 13, 2000 (Igaliku; data provider: DigitalGlobe).

Fossil, raised beach ridges have been identified and described in several places near the present Greenlandic coastlines with evidence of significant relative sea-level changes during the Holocene^4^,^6^,^12^. Well-developed beach ridge systems at Tuapaat, Qassiarsuk and Igaliku are investigated in this study (Fig. 2a–c). The beach ridge system at Tuapaat is well-exposed and readily observable on maps. The systems at Qassiarsuk and Igaliku are partly exposed and partly masked by vegetation.

In geomorphological terms, Tuapaat in western Greenland is categorized as a cuspate foreland, and the fossil beach ridges found at this locality consist of coarse grained clastic material^6^. Freezing and thawing processes have reworked and deformed the surface morphology and to some extent changed the geomorphological appearance of the beach ridges^6^. The highest Holocene marine limit at Tuapaat is at about 80 m above sea-level^6^.

Beach ridge systems in southern Greenland have been mapped in previous studies, and the highest Holocene marine limit at Qassiarsuk and Igaliku have been estimated to be 50 m and 53 m, respectively^4^. These beach-ridge systems are also gravelly with typical clast sizes of more than 5 cm, similar to Tuapaat. Isolation basin-based studies from southern Greenland have dated Holocene sea-level indicators to up to 15 kyr^10^,^11^.

## Results

The surveys consisted of a combination of GPR reflection recordings and topographic measurements with a differential GPS (Trimble RTK-R8 DGPS) system following the same protocol as Hede *et al*.^26^. The topographic DGPS measurements were supplemented with topographic variability imaged by kite aerial photography^29^ (Fig. 2d,e) at the two locations (Igaliku and Qassiarsuk) in South Greenland. The kite aerial topography data clearly delineate the detailed surface topography, and constitute an important aid for outlining the areal extent of the beach ridge systems at Igaliku and Qassiarsuk.

### Description of GPR data

**Figure 3:**
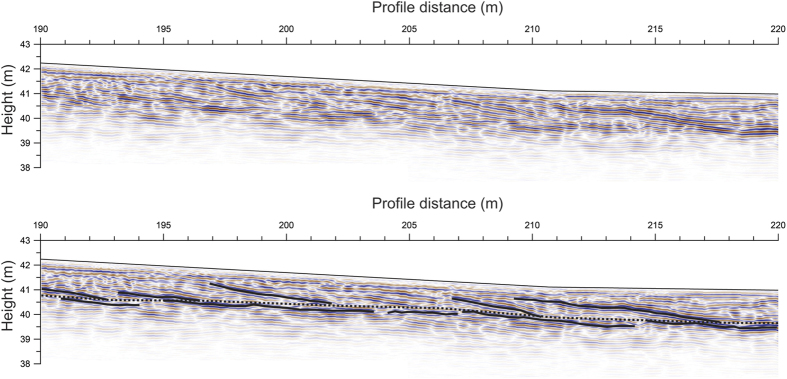
Section of ground-penetrating radar (GPR) section collected on elevated beach ridge plain at Tuapaat without (top) and with (bottom) interpretation. Examples of reflections showing dips characteristic for interpreted beachface and upper shoreface deposits have been highlighted with bold lines and the separation between beachface and upper shoreface deposits is shown with a dotted line. See text for interpretation. Trace spacing is 0.05 m. At each trace location a total of 8 measurements were made and stacked to improve the signal-to-noise ratio. Data section migrated using REFLEXW software.

**Figure 4:**
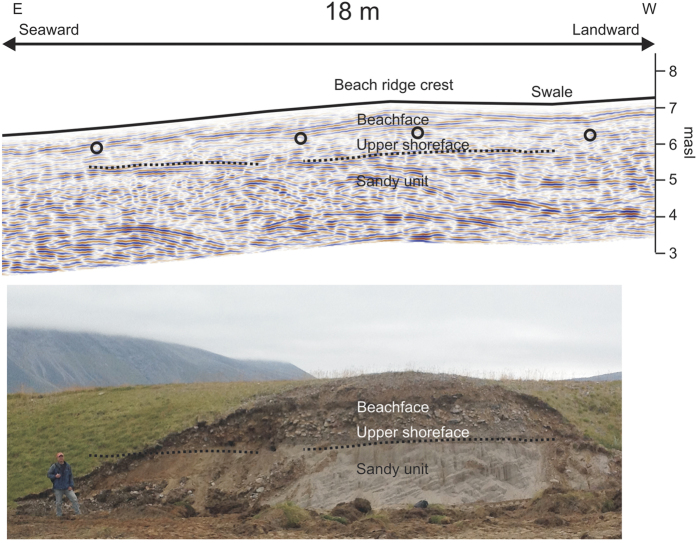
Outcrop of beach ridge eroded by river (bottom) and GPR reflection profile collected ~25 behind this outcrop (top) (see Fig. 2 for location). Circles indicate interpreted downlap points marking transition from beachface to upper shoreface. Dotted lines show GPR reflections that are interpreted to show transition from upper shore face coarse-grained sediments to the sandy layer below. These dotted lines have been copied onto the photograph below. Note that the sandy unit shows rather steep seaward-dipping layers in the outcrop. These features are consistent with relatively weak dipping events in the eastern part of the corresponding GPR image, but they do not constitute a general feature of the GPR image at depths representative of the sandy unit. Thus, the steep seaward-dipping events are not a general feature of the deeper sandy unit. GPR data section migrated using REFLEXW software. Person on photograph is Lars Nielsen. Photographer: Aart Kroon.

**Figure 5:**
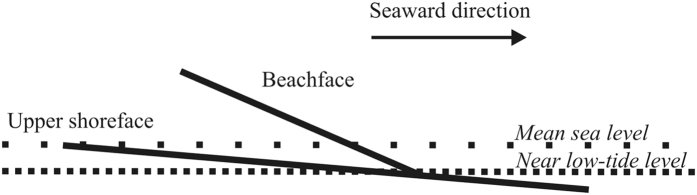
Conceptual model for position of downlap point with respect to sea-level. Due to the apparent high energy level under which the beach ridge systems have been deposited (coarse-grained matrix), we assume that the downlap point marking the boundary between interpreted beachface and upper shore face deposits is located near the low-tide level.

The surface topography of the beach ridge plains shows alternating positive structures (beach ridges) and negative structures (swales). The examples of Figs 3, 4 and 5 show 5–20 m wide ridges with an amplitude of typically up to about 1 m.

The collected GPR data sections show continuous reflection patterns to a depth of 3–4 m, where reflection amplitudes become relatively weak due to the effects of geometrical spreading, transmission losses and absorption^30^. The GPR sections generally show clear, continuous seaward-dipping (generally ~5° to ~10°) reflections in the uppermost 1–2 m of the subsurface (Fig. 3 and Supplementary Material). Undulating parallel to sub-parallel reflections with smaller dips are observed below these dipping reflections. The transition between these two packages of reflectivity is often clearly marked by the termination of the seaward-dipping reflections from the upper reflectivity package (shown by dotted lines in Fig. 3 and Supplementary Material). These general observations resemble observations made across beach ridge systems in the micro-tidal environment of southwest Scandinavia^20^,^23^,^25^,^26^ and in Japan^16^. This characteristic picture is at times disturbed by more chaotic reflection patterns characterizing the uppermost 1 m (see Supplementary Material).

### Interpretation of GPR reflectivity

At Igaliku, a river has cut through a part of the studied beach ridge plain and left internal beach ridge structures and upper shoreface deposits exposed in the field (Fig. 4). Furthermore, and older sandy unit is visible below the upper shoreface deposits. At this location, the maximum clast size of the upper shoreface and beachface deposits is about 0.4 m in diameter, and the stones that make up the main layering are irregular to rounded in shape, indicating different maturation levels. The matrix of the beach ridge shown in Fig. 4 is primarily sand; however, the sand content is highly variable at Igaliku, and sand is not always present in the matrix. Internal beach ridge GPR reflections represent clast-rich layers that form downlap points interpreted to mark transition from beachface to upper shoreface deposits. Moreover, the transition from the upper shoreface deposits to deeper sandy strata is also clearly imaged by a reflection event, although this level appears discontinuous at one point in the GPR reflection image, most likely due to interference with (multiple) reflections from internal beach ridge and upper shoreface structures. Thus, there is good consistency between GPR reflectivity and exposed structures. However, it should be noted that the GPR reflection image of Fig. 4 is collected ~25 m behind the exposed wall, and a strict correlation between outcrop exposures and GPR reflectivity is therefore not possible.

The observed reflection patterns observed at the different Greenlandic study sites are interpreted to represent relatively steeply dipping beach face deposits on top of less steeply dipping upper shoreface deposits, consistent with previous interpretations from other settings^16^,^23^,^26^. One exception from this general pattern is at the exposed beach ridge at Igaliku presented in Fig. 4. At this particular location, the sandy unit below the coarser-grained beach ridge and upper shoreface deposits exhibits steep-dipping strata in the outcropping exposure. However, by comparison to the approximately coincident GPR image, it is clear that this is not a general feature, because the GPR image shows different dip directions for the depth levels representative of the sandy unit. Moreover, the GPR reflectivity corresponding to the deeper sandy unit predominantly shows landward-dipping events and only to some extend seaward-dipping events, whereas the exposed sand predominantly shows seaward dips. Besides, the boundary separating the beachface and upper shoreface deposits is expected to represent a level close to or at sea-level at the time of deposition^16^,^23^,^26^. In a few places, two such levels may be identified on top of each other (see Supplementary Material) suggesting deposition of two generations of upper shoreface deposits. The level where steeply dipping beach deposits downlap on to less-dipping upper shoreface deposits has been interpreted to correspond to about 1 m below mean sea-level at the beach ridge plain at the Pacific coast of Japan^16^. This level corresponds approximately to the low-tide level in that area. Tamura *et al*.^16^ constrained their interpretation of GPR reflection images by correlation to a geologically interpreted borehole and sea-level observations. Similar reflection patterns observed in GPR reflection sections for three different localities in the micro-tidal regime of southwest Scandinavia correspond to actual sea level at the time of deposition^20^,^23^,^26^. The three Greenlandic study sites investigated here are all located in meso-tidal regimes according to data from 2016 of the Danish Meteorological Institute. Moreover, the coarse-grained material that constitutes the matrix of the studied beach ridges indicates a high average energy level during deposition. We consider that the downlap points observed here represent a level close to the low-tide level at the time of deposition due to an expected higher preservation potential for sediments deposited near that level (Fig. 5).

The more chaotic patterns sometimes disturbing the upper ~1 m of these general reflectivity patterns (see Supplementary Material) are interpreted to be evidence of freezing/thawing processes or erosion processes that have been in effect since the deposition of the beach ridge deposits. Clearly, such secondary processes perturb beach ridge morphology. However, they do not always reach deep enough to disturb the boundary marking the transition between beachface and upper shoreface that typically is situated about 1 to 1.5 m below the ridge surface (see Supplementary Material). Moreover, the alterations caused by the interpreted freeze-thaw processes may not be readily observable in the surface morphology alone.

## Discussion

The first GPR images of beach ridge deposits from different raised beach plains/systems in Greenland are presented here. The three study sites investigated here represent areas where the surface morphology of the beach ridge systems is exposed to varying extent. However, GPR profiling makes imaging of the internal beach ridge structures possible at all three locations. Proper imaging of the beach ridge structures requires careful migration of the recorded reflection section due to significant scattering caused by the relatively coarse-grained material that typically constitutes the main part of the studied beach ridge systems. However, the final processed data facilitate interpretation of internal beach ridge system layering and structure with the same degree of resolution as obtained at other study sites in temperate regions, where beach ridge systems are composed of finer-grained mixed sand and gravel^19^,^20^,^26^.

The GPR data sets are interpreted to show seaward-dipping prograding beachface deposits developed on top of upper shoreface deposits with shallower dips. Moreover, areas influenced by shallow freeze/thaw processes and surface deformation and erosion are identified, and the transition between beachface and upper shoreface deposits may be identified underneath such areas. Thus, the presented approach has the potential to also provide trustworthy estimates of relative sea level in areas where traditional studies of beach ridge morphology^12^,^13^ cannot provide precise estimates of relative sea level. Parts of the Holocene deposits may be situated below present sea level^11^,^27^, and such submerged deposits cannot be studied with the GPR technique. Thus, for some areas, a complete, continuous Holocene sea-level curve cannot be established using the proposed methodology alone. However, the old and youngest (<2 kyrs) should generally always be accessible, and for those times high-resolution relative sea-level curves should be possible to obtain. Moreover, the most recent deposits may be the most critical to study for improved understanding of the impact of present climate change.

The overall internal beach ridge layering and structure is very similar to beach ridge systems deposited under micro tidal conditions in inland seas of Denmark, although the Greenlandic beach deposits are expected to have formed under meso-tidal conditions as judged from modern day conditions. The exact relation between identified internal markers and sea level depend on different factors, such as tidal range, wave action, and sediment supply. While Tamura *et al*.^16^ interpreted similar changes in GPR reflectivity patterns to corresponded to a level of c. 1 m below sea level at a locality on the Pacific coast of Japan, Nielsen & Clemmensen^23^ showed that such features identified in GPR data collected on Anholt, central Kattegat Sea between Denmark and Sweden, exhibit a scatter around mean sea level at the time of deposition, which primarily reflects meteorologically induced changes in water levels in that area where the tidal range is less than 0.4 m. For a different locality in southeastern Denmark, Hede *et al*.^26^ found results that are similar to the ones of Nielsen & Clemmensen^23^. The sea-level markers interpreted from the GPR data collected at Tuapaat, Qassiarsuk and Igaliku exhibit a relatively small scatter as compared to the tidal range of about 3 m in these areas. Besides, the downlap points observed in the GPR sections mainly exhibit long-wavelength variation and short-scale fluctuations are not dominant. These observations indicate that the sea-level markers observed in these GPR sections were formed systematically with respect to sea level at the time of deposition and that here the internal beach ridge structures have only been moderately affected by variation in elevated water levels due to tides and/or meteorological factors. Moreover, observations made in GPR profiles acquired near the present coast line are consistent with the interpretation that the identified level separating steeply seaward-dipping reflectivity from less dipping features represents a height close to sea level. Further, the dominant clast size of the beach ridge systems and the tidal range of the different localities are comparable suggesting that the downlap points of the different localities most likely refer to the same level, probably close to the low tide sea level^16^ (see also conceptual model of Fig. 5). These similarities suggest that the relative sea-level variations that may be extracted from the presented approach will be directly comparable and may form a solid basis for studying regional variations in sea level and vertical land movement during the Holocene. Our mapping is consistent with the previous results by others showing tens of metres of relative sea-level change in southern and western Greenland indicative of large isostatic rebound effects in relation to glacial melting and retreat^5^,^6^,^10^,^11^.

The presented GPR-based approach allows for identification of numerous sea-level markers in the fossil beach deposits. An age model is the next step for the study sites, but clearly the resolution of the variability of the relative sea-level curves to be established based on such studies will be limited mainly by the availability on dateable material, e.g. material suitable for optically stimulated luminescence dating^31^ or organic matter for ^14^C dating^27^ and not the availability of sea-level markers.

The beach ridge deposits studied here cover limited geographical areas of a few square kilometers (Fig. 2) and probably represent several thousand years of deposition^4^,^6^,^9^,^27^. The melting of ice sheets in Greenland has resulted in strong differential vertical land movement, which influences sea-level curves extracted from markers integrated over large areas^10^,^11^. The application of the presented methodology can result in relative sea-level curves that are consistent with respect to the influence of vertical land movement since the individual beach ridge plains are so spatially localized that they have not experienced spatially varying uplift histories during their evolution. Therefore, we only expect variation in uplift with time for the individual beach ridge plains.

## Methods

The topographic data was collected along profile lines and on specific points with two RTK differential GPS systems, one served as basis on a fixed point in each study site, while the other served as rover. The data of both identical differential GPS systems were combined and post-processed using Trimble software. The accuracy of the final location of the individual points was in the order of 0.05 m (horizontal) and 0.05–0.1 m in height (vertical).

The GPR data was collected along profile lines oriented approximately perpendicular to the orientation of the beach ridges using shielded 250 MHz antennae manufactured by Sensors & Software (Fig. 2). Eight measurements, which were subsequently stacked to one data trace, were acquired every 0.05 m along the profile lines. Data was subject to standard processing including: dewow, gain corrections, band-pass filtering, migration and depth conversion^20^,^26^. The migration algorithm accounted for beach ridge and swale topography^32^. Migration and depth conversion relied on estimation of GPR wave velocities. Inspection of diffraction hyperbolas observed in the non-migrated record sections showed that the root-mean-square (rms) velocity varied between 0.07 and 0.12 m/ns at all three localities. Large round stones with a diameter of 5 cm or more often constituted the main matrix of the studied beach ridges. Such stones were often large enough to cause significant scattering of the GPR wavefield. Therefore, careful rms-velocity estimation and migration were particularly essential processes for obtaining high-resolution reflection images of the subsurface layering. The frequency spectrum for the recorded data typically shows a fairly broad peak from ~120 MHz to ~300 MHz. The vertical resolution of the obtained reflection images is about 0.1 m or better for the typical dominant wavelength of the data. The uncertainty of individual depth estimates to reflectors is around 0.25 m taking errors related to topographical corrections, velocity uncertainties and possible misidentification of reflections (due to e.g. precursors caused by frequency filtering) into account^23^. The migration and depth conversion made for the GPR image shown in Fig. 4 was made for a velocity of 0.07 m/ns. Thus, for the central frequency of ~250 MHz, the wavelength of the GPR signal is ~0.3 m at this location. For this wavelength, the stony/rocky layers of the interpreted beach ridge and upper shoreface deposits appear as almost continuous, smooth layers in the GPR data reflection image (Fig. 4).

## Additional Information

**How to cite this article:** Nielsen, L. *et al*. Sea-level proxies in Holocene raised beach ridge deposits (Greenland) revealed by ground-penetrating radar. *Sci. Rep.* **7**, 46460; doi: 10.1038/srep46460 (2017).

**Publisher's note:** Springer Nature remains neutral with regard to jurisdictional claims in published maps and institutional affiliations.

## Supplementary Material

Supplementary Material

## Figures and Tables

**Figure 1:**
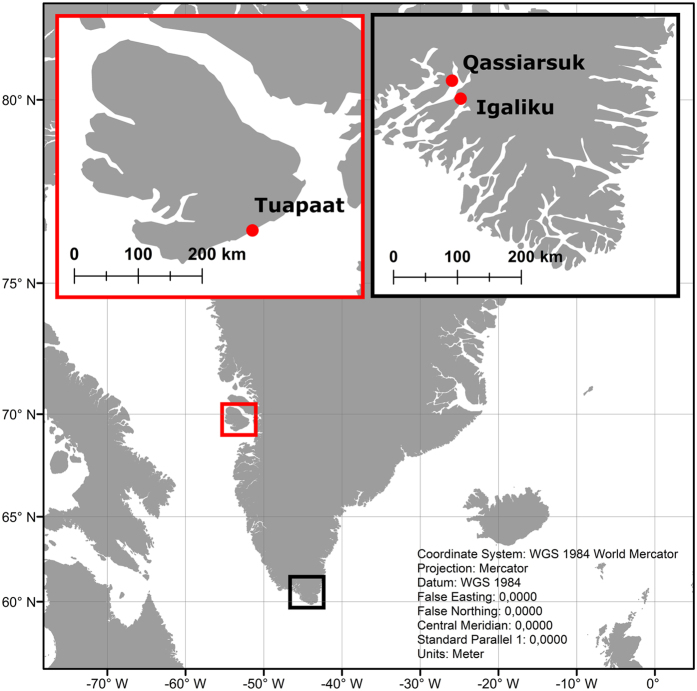
Maps showing location of study sites in South and West Greenland with red dots. Inset maps of Greenland show the regions where the study sites are located (boxes). Made with Natural Earth. Free vector and raster map data@naturalearthdata.com.
