# Supplementary material for: Sea-level proxies in Holocene raised beach ridge deposits (Greenland) revealed by ground-penetrating radar
Source: Sci Rep. 2017 Apr 19;7:46460. doi: 10.1038/srep46460 (PMC5396070; doi:10.1038/srep46460)
Supplement: Supplementary Material [file srep46460-s1.pdf]

Sea-level proxies in Holocene raised beach ridge deposits (Greenland) revealed by ground-penetrating radar

Lars Nielsen[1*], Mette Bendixen[1,2], Aart Kroon[1,2], Mikkel Ulfeldt Hede[1**], Lars B. Clemmensen[1], Ronny Wessling[3], Bo Elberling[1,2]

[1]Department of Geosciences and Natural Resource Management, University of Copenhagen, Øster Voldgade 10, 1350 Copenhagen K, Denmark

[2]Center for Permafrost (CENPERM), Department of Geosciences and Natural Resource Management, University of Copenhagen, Øster Voldgade 10, 1350 Copenhagen K, Denmark

[3]Department of Prehistoric and Historical Archeology, University of Vienna & Crazy Eye, Geoinformatics and Digital Archaeology

*Correspondence to ln@ign.ku.dk

**Now at: Tårnby Gymnasium og HF, Tejn Alle 5, 2770 Kastrup, Denmark.

Supplementary Material

In this supplementary material, two additional GPR lines collected at Qassiarsuk (Fig. 5) and Igaliku (Fig. 6) are given.

Fig. 6. Section of ground-penetrating radar (GPR) section collected on elevated beach ridge plain at Qassiarsuk (Fig. 2a) without (top) and with (bottom) interpreted level separating beachface and upper shoreface deposits. See main text for interpretation. Trace spacing is 0.05 m. At each trace location a total of 8 measurements were made and stacked to improve the signal-to-noise ratio. Data section migrated using REFLEXW software.

Fig. 7. Section of ground-penetrating radar (GPR) section collected on elevated beach ridge plain at Igaliku (Fig. 2b) without (top) and with (bottom) interpreted level separating beachface and upper shoreface deposits. Note chaotic patterns in the upper ~1 m between ~2-12 m profile interpreted to be effects of freezing/thawing processes .. Trace spacing is 0.05 m. At each trace location a total of 8 measurements were made and stacked to improve the signal-to-noise ratio. Data section migrated using REFLEXW software.

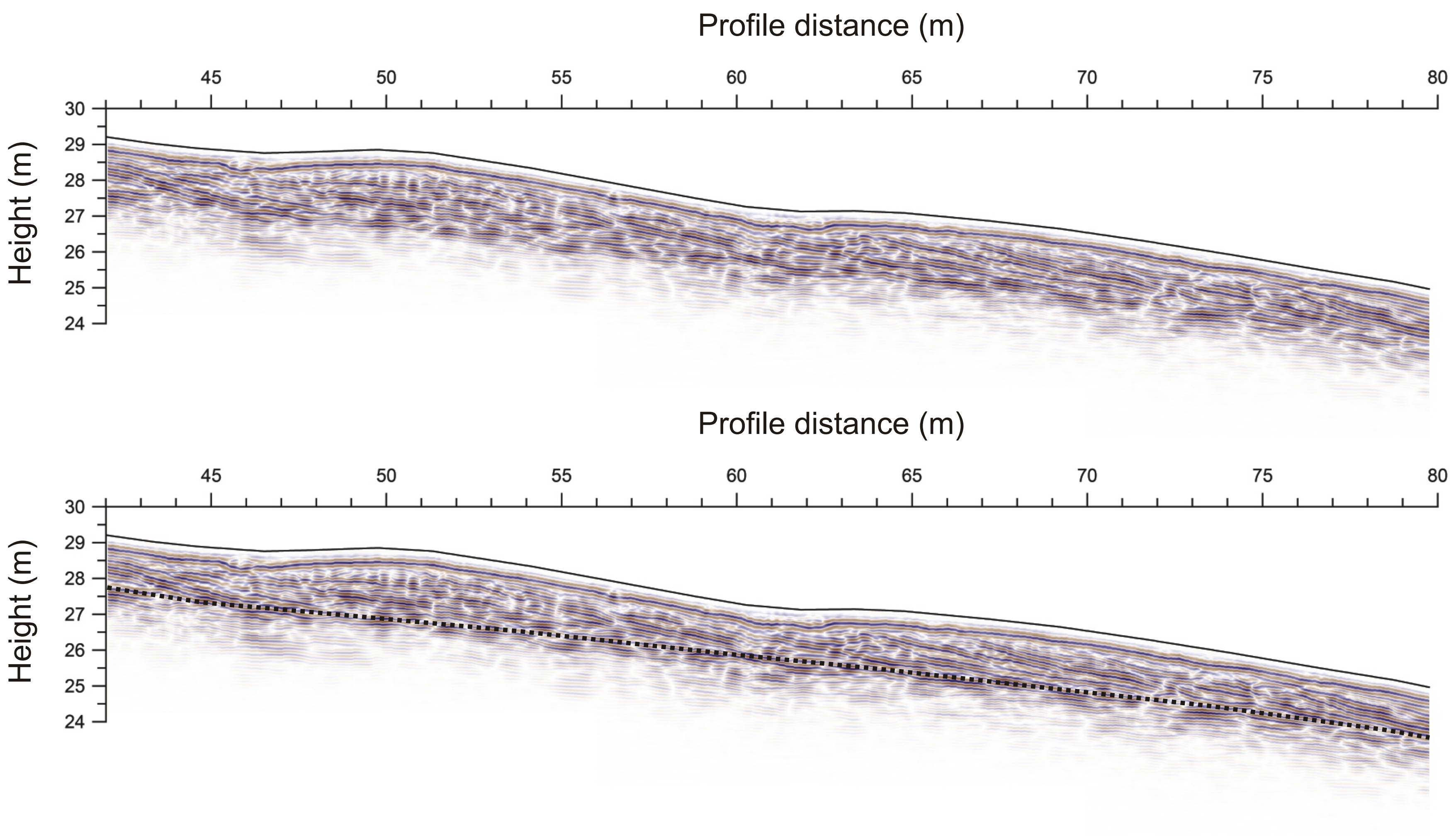
Profile distance (m)
45
50
55
60
65
70
75
80
Height (m)
30
29
28
27
26
25
24
Profile distance (m)
45
50
55
60
65
70
75
80
Height (m)
30
29
28
27
26
25
24

Fig. 6

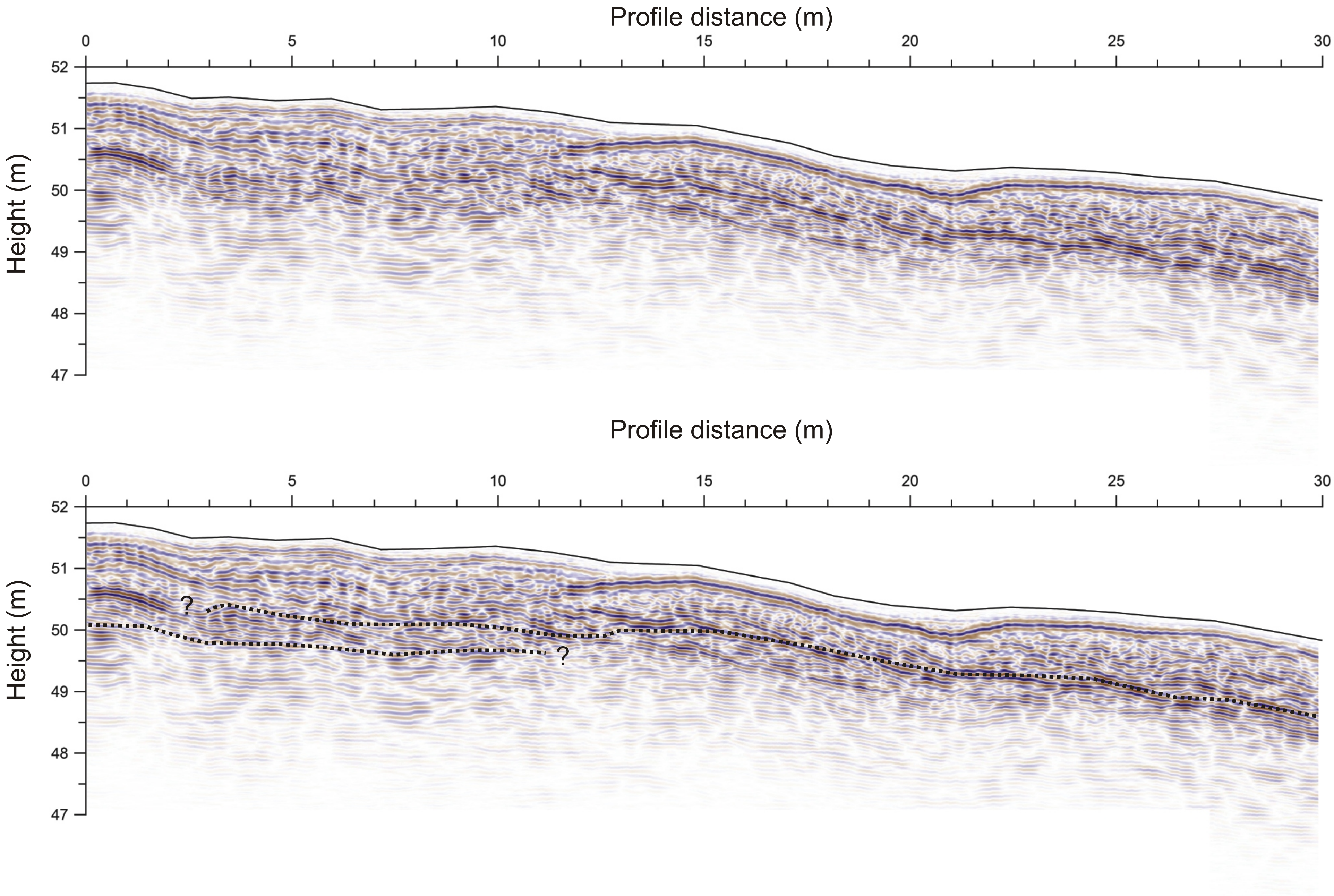
Profile distance (m)
0
5
10
15
20
25
30
Height (m)
52
51
50
49
48
47
Profile distance (m)
0
5
10
15
20
25
30
Height (m)
52
51
50
49
48
47
?
?

Fig. 7
